# A rare case of pseudoaneurysm at the site of untreated coarctation of the aorta in an adult patient

**DOI:** 10.1007/s11748-020-01525-w

**Published:** 2020-10-28

**Authors:** Takuma Mikami, Takeshi Kamada, Hiroki Uchiyama, Yosuke Kuroda, Ryo Harada, Syuichi Naraoka, Nobuyoshi Kawaharada

**Affiliations:** Department of Cardiovascular Surgery, Sapporo Medical University School of Medicine, 291, Minami 1-jo Nishi 16-chome, Chuo-ku, Sapporo, Hokkaido 060-8543 Japan

**Keywords:** Coarctation of the aorta, Pseudoaneurysm, Adult congenital heart disease

## Abstract

Here we report a rare case of pseudoaneurysm at the site of aortic coarctation. Aortic coarctation and a saccular aortic aneurysm protruding from the site of this coarctation were detected in a 50-year-old woman. Owing to the shape of the aneurysm and high risk of rupture, an open surgical repair was performed. The pathological findings of the removed aneurysm revealed a pseudoaneurysm consisting of only a thin adventitial wall. Adult uncorrected aortic coarctation has a poor prognosis. One of its causes may be the formation of such a pseudoaneurysm.

## Introduction

Coarctation of the aorta (CoA) has been reported to account for 4–8% of congenital heart diseases [1–3]. There are few rare cases of detected CoA in adults, which was not detected earlier during childhood, and the natural course of the disease is complicated due to cardiac malformations and cardiovascular comorbidities [2]. Few cases have been reported on the association between pseudoaneurysms and CoA. However, these cases were reported due to post-stenotic dilation. To the best of our knowledge, there have been no reports of pseudoaneurysm at the stenotic site yet. Here, we present a rare case of a patient with CoA who was asymptomatic until the age of 50 and had a pseudoaneurysm protruding from the site of aortic coarctation. We decided to perform an open surgical repair. The pathological findings of the resected aneurysm wall revealed a saccular pseudoaneurysm comprising only an adventitia. Because of the very thin aneurysm wall, the risk of aneurysm rupture was extremely high. Therefore, it is thought that the pseudoaneurysm rupture as in this case may be one of the causes of the poor prognosis of adult aortic coarctation.

## Case

**Fig. 1:**
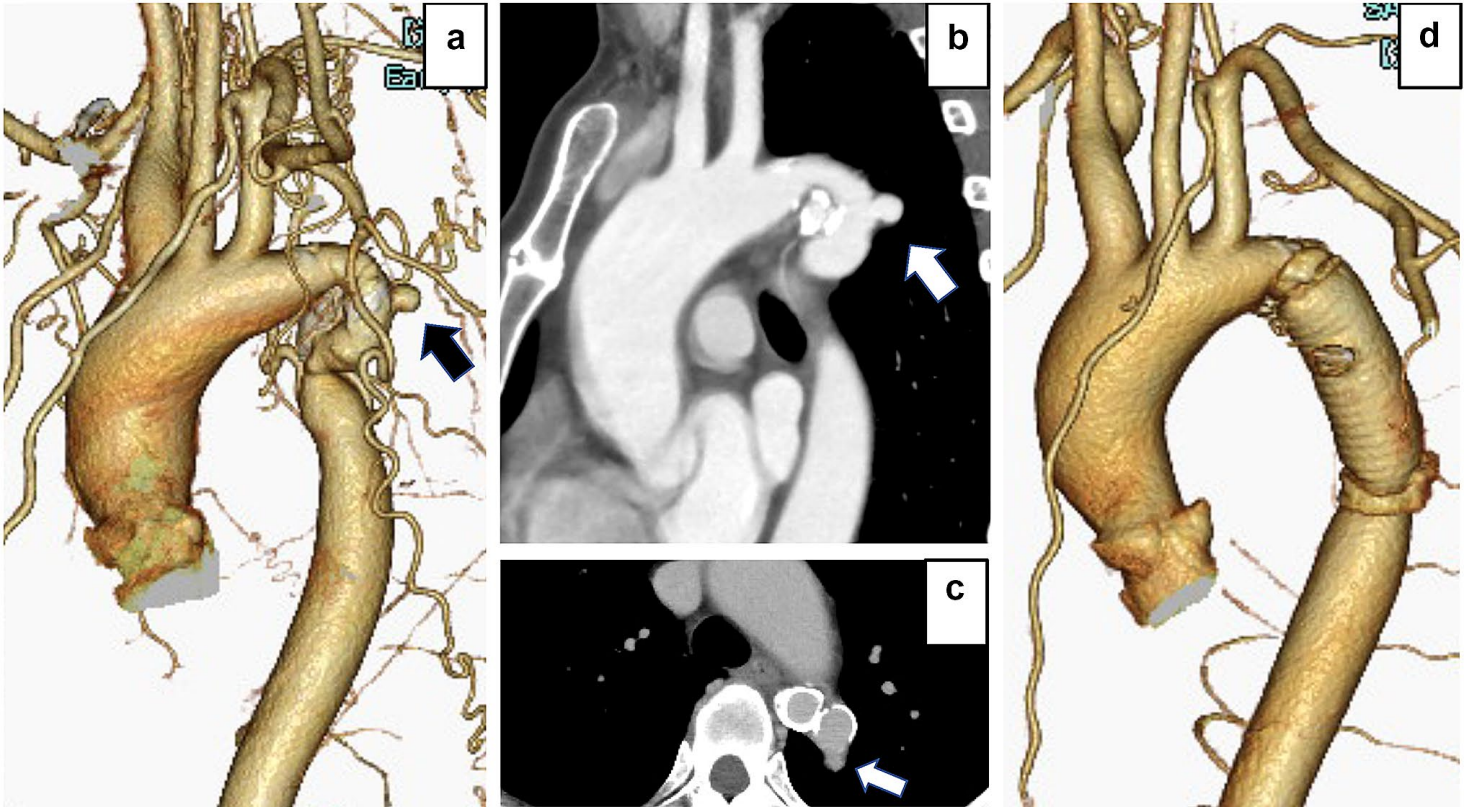
**a** Preoperative contrast-enhanced CT scan: a saccular aortic aneurysm is observed at the site of aortic coarctation (arrow). Collateral circulation appears well developed. **b** Preoperative contrast-enhanced CT scan, sagittal section: a saccular aortic aneurysm protruding dorsally is observed. The aneurysm wall appears very thin (arrow). **c** Preoperative contrast-enhanced CT scan, axial section: a dorsally protruding saccular aortic aneurysm is observed, the arterial wall other than the aneurysm appears highly calcified. **d** Postoperative contrast-enhanced CT scan: the stenosis was completely resected

A 50-year-old woman visited a nearby physician for a medical checkup due to complaints of high blood pressure. An ankle brachial index (ABI) showed a pressure difference between the upper and lower extremities. A computed tomography scan (CT) showed CoA and a saccular aortic aneurysm protruding from the aortic coarctation. In addition, she had untreated dyslipidemia. The ABIs of the right and left ankles were 0.65 and 0.67, respectively. Transthoracic echocardiographic showed no significant valvular dysfunction or left ventricular remodeling due to heart failure. Notably, no combined vascular malformations were observed. The CT scan showed that CoA originated from the periphery of the left subclavian artery to the aortic isthmus, and classified as a CoA with isthmus hypoplasia [4]. In addition, a saccular aneurysm protruding dorsally from the coarctation was observed (Fig. 1a, b). Calcification in the saccular aneurysm wall was not observed. However, high calcification in the aortic area except for the aneurysm was observed (Fig. 1c). No other abnormalities were observed in the heart or large vessels. Laboratory data showed that erythrocyte sedimentation rate and C-reactive protein levels were within the normal reference ranges. Considering the high risk of aneurysm rupture, we decided to perform an open surgical repair. Written informed consent was obtained from the patient to publish this case report.

**Fig. 2:**
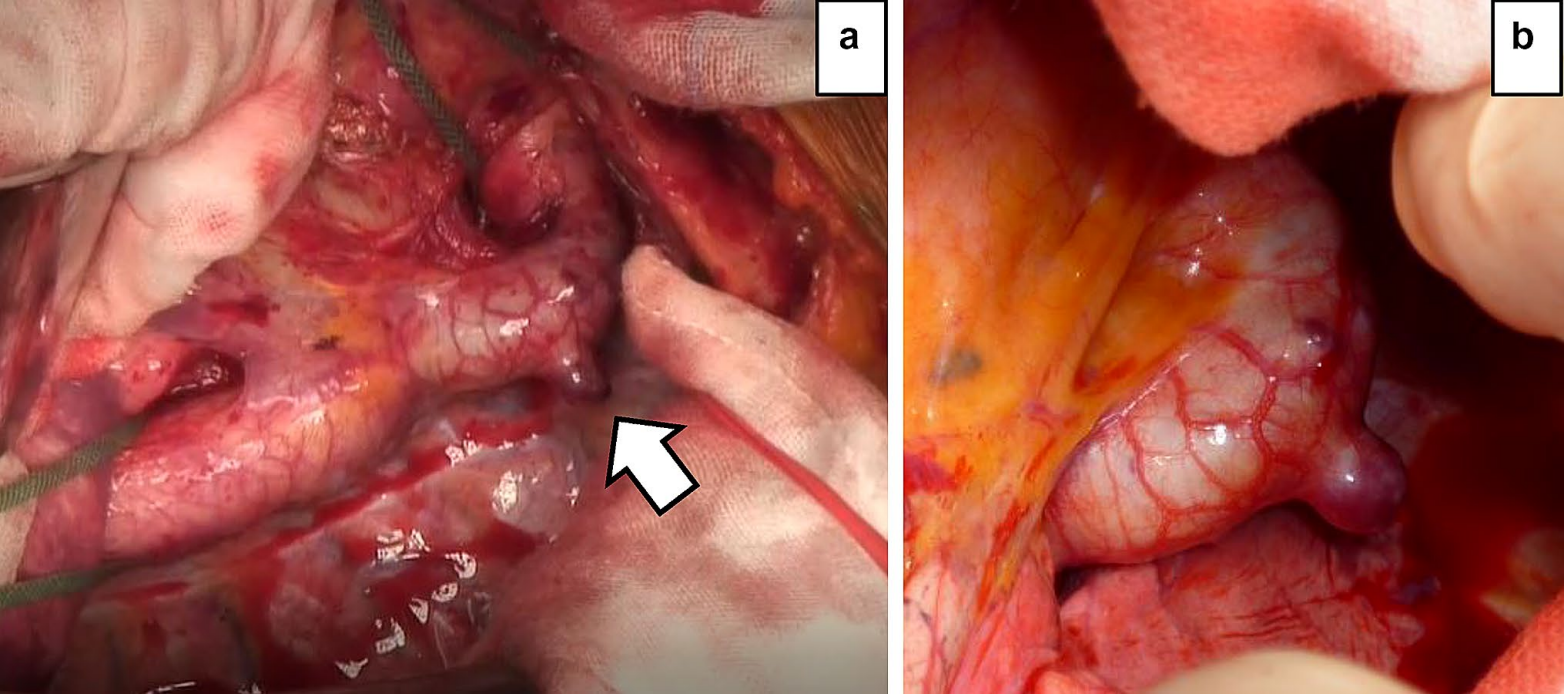
**a** Intraoperative image: a saccular aortic aneurysm protruding dorsally at the coarctation site (arrow). **b** Enlarged image: the aneurysm wall appears thin

**Fig. 3:**
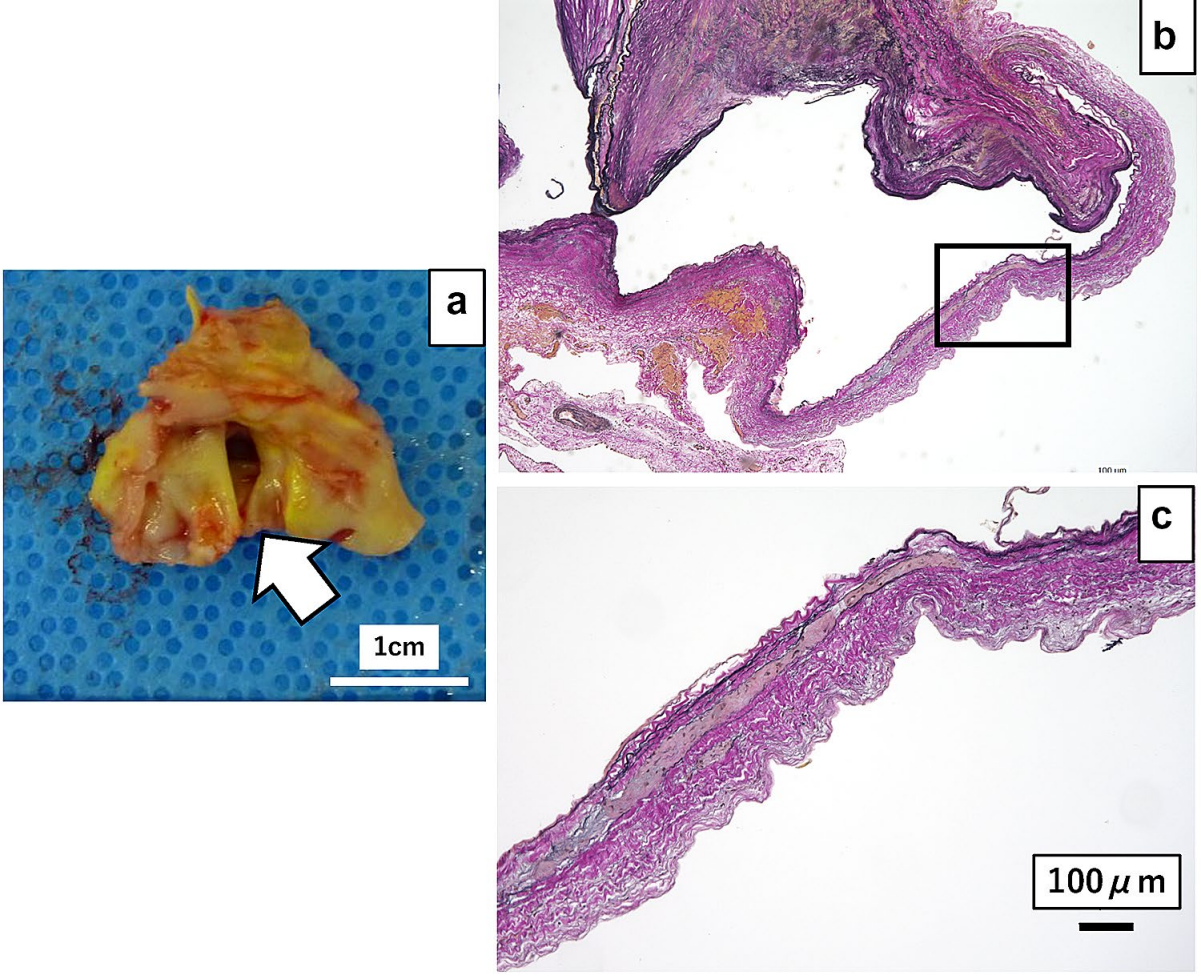
**a** A photo of the resected aneurysm from the intima side: the intima is macroscopically missing at the aneurysm entrance (arrow). **b** Elastica van Gieson staining of the aneurysm wall: the aneurysm wall has a clearly thinner wall structure than the other parts. **c** Enlarged findings (within the square): histologically, the intima and media are absent. Several layers of elastic fibers were found (arrow); therefore, a pseudoaneurysm consists of the adventitia only

Thoracotomy was performed under general anesthesia via the left lower 6th intercostal. No adhesion between the aneurysm and the lung was found. We clamped the aorta between the left common carotid and left subclavian arteries. Therefore, anastomosis was performed by clamping the central side of the descending thoracic aorta at the Th8 level without deep hypothermic circulatory arrest. This was performed more proximal than the segmental artery connecting to the Adamkiewicz’ artery (AKA) identified preoperatively at the left Th9 level. The right common femoral artery and vein were cannulated, and partial cardiopulmonary bypass was established during aortic clamping. The aneurysm mass atrophied easily after clamping (Fig. 2). The saccular aneurysm lacked a defined intima and was formed of a thin single-layered membrane, which was indicative of the adventitia (Fig. 3a). The anastomosis was performed with a 20-mm 1-branch tubular graft (J Graft SHIELD NEO^®^, Japan Lifeline Co., Ltd. Tokyo, Japan). A drain was placed in the left thoracic cavity. The operative and cardiopulmonary bypass time were 194 and 58 min, respectively. In addition, the aortic block time, minimum body temperature and bleeding volume were 54 min, 34.8 °C, and 590 mL, respectively.

Histopathologic examination of the excised specimen showed the presence of several layers of elastic fibers, but no intimal or medial structures were observed in the saccular pseudoaneurysm. No acute or chronic inflammation was present. Therefore, these findings, clearly revealed a pseudoaneurysm consisting of the adventitia only (Fig. 3b, c).

The patient’s postoperative course was uneventful. Mild chylous effusion was observed from the left thoracic drain. Improvement was achieved with a conservative treatment (with a low-protein diet), and the drain was removed 7 days after surgery. A postoperatively contrast-enhanced CT scan revealed a complete coarctation resection, and no abnormal findings were observed in the artificial blood vessel anastomosis (Fig. 1d). Improvements were observed in postoperative ABI (right ABI 0.82, left ABI 0.84). The patient was discharged 23 days postoperatively.

## Discussion

Many cardiac malformations are associated with CoA [5]. Various factors, such as genetic, environmental, and arterial plaque formation are involved in the pathophysiology of CoA. Cases of adults with CoA that have remained undetected during childhood are rare, and are often asymptomatic or hypertensive. The natural history of patients with CoA is poor. The average survival age of patients with untreated CoA beyond childhood is 34 years, with 75% mortality by age 43 years [2]. The causes of death include heart failure, aortic dissection or rupture, infective endocarditis, and intracranial hemorrhage due to ruptured cerebral aneurysm [6]. Very rare undiagnosed cases (such as the present case) survive without treatment until the age of 50 due to a well-developed collateral circulation from the internal thoracic and intercostal arteries. Peripheral ischemia symptoms were not identified from the coarctation. There have been some reports on the complications of CoA and aortic aneurysms. The etiological causes of pseudoaneurysm formation include: arteriosclerosis due to hypertension; arterial wall weakness due to inflammation (infectious endocarditis, aortic inflammation); congenital abnormalities in the arterial wall structure and post-stenosis dilation; and incompletely occluded arterial ducts [7]. Yet, there are few reports on the association between pseudoaneurysms and CoA. Oi et al. [8] and Prifti et al. [9] reported that an aneurysm was formed at the periphery of the coarctation, and attributed to a jet stream of aortic stenosis. In addition, the location of aneurysm was more peripheral than that of stenosis. However, in this present case, a pseudoaneurysm was formed in the aortic constriction, and severe calcification was detected around the same area. The pathological findings showed that the intima and media at the site of coarctation were completely unobserved and the very thin aneurysm had formed a few layers of elastic fibers. These findings also suggested that the pseudoaneurysm had a very high risk of rupture, and this is one of the causes of poor progression of coarctation of aorta in adults.

In CoA, symptomatic or asymptomatic, the difference in blood pressure between the upper and lower limbs (at rest) was 20 mmHg or more. In addition, hypertension or left ventricular hypertrophy were indications for class I surgery according to the European and American guidelines. A class IIa surgery was considered in individuals even when the maximum pressure difference was 50% or more [10, 11]. In this case, the difference in blood pressure between the upper and lower limbs ranged approximately between 40 and 50 mmHg that was complicated by hypertension. Surgical procedures included end-to-end anastomosis, patch formation, subclavian flap aortoplasty, and artificial blood vessel replacement [12]. In the recent years, treatment using thoracic endovascular aortic repair (TEVAR) has been reported. However, in this present case, extensive calcification of aortic constriction area and complications were observed in the pseudoaneurysm. Therefore, end-to-end anastomosis, patch formation, subclavian flap aortoplasty, and TEVAR were considered as off-label treatments, and artificial blood vessel replacement was selected. These findings suggest that a complete resection of the stenosis including calcification and artificial blood vessel replacement are appropriate procedures in cases of CoA with advanced calcification. However, the findings cannot be generalized due to the limited number of cases and further studies are required to validate our findings.

## Conclusion

We studied a rare case of a pseudoaneurysm at the site of aortic coarctation. The natural prognosis of untreated CoA is poor, and pseudoaneurysm at the site of aortic coarctation may be one cause of poor prognosis.
